# Low-Speed Permanent Magnet Synchronous Motor Rotor Position Estimation Using Structural Vibration Modal Phase Carrier

**DOI:** 10.3390/s26051707

**Published:** 2026-03-08

**Authors:** Linxin Yu, Xin Yuan, Jing Ou

**Affiliations:** 1 School of Electrical Engineering and Automation, Shenyang Ligong University, Shenyang 110159, China; yulinxin@sylu.edu.cn; 2 School of Electrical Engineering and Automation, Harbin Institute of Technology, Harbin 150001, China; jing.ou@hit.edu.cn

**Keywords:** permanent magnet synchronous motor, structural vibration, modal phase, rotor position estimation, low-speed operation

## Abstract

To address the challenges of diminished back-EMF, high noise interference, and reduced accuracy in traditional low-speed sensorless control, this study proposes a rotor position estimation method based on structural vibration characteristics. The coupling mechanism between air-gap electromagnetic force density and stator structural vibration modes is analyzed. This analysis reveals that rotor spatial information is embedded within specific modal phases, establishing the physical basis for utilizing vibration phase as a position carrier. Accordingly, a workflow encompassing signal acquisition, modal selection, and phase calculation is developed and integrated into a sensorless control system. Simulation results demonstrate that the proposed method achieves stable estimation even under strong noise. The estimation error shows clear performance advantages over conventional back-EMF-based methods in the low-speed region, validating its effectiveness and robustness at low speeds. This research provides a new approach that introduces non-electrical structural information as a complementary channel to overcome the inherent limitations of electrical-signal-based position estimation at low speeds.

## 1. Introduction

Permanent magnet synchronous motors (PMSMs) are widely used in electric vehicles, industrial servo systems, and marine propulsion applications due to their superior performance characteristics, including high power density, high efficiency, and low noise. In high-performance drive systems, rotor position information is a critical parameter for achieving field-oriented control and high dynamic response. To reduce system costs and enhance operational reliability, sensorless control technology has emerged as a major research focus globally [1,2]. However, existing PMSM rotor position estimation methods exhibit poor adaptability across different salient-pole motor types and are susceptible to electromagnetic parameter fluctuations. Consequently, improving the robustness of sensorless control and optimizing rotor position estimation accuracy have become critical research directions in motor control [3,4].

Rotor position self-detection control methods based on back EMF [5,6] achieve relatively accurate estimates in medium-to-high speed ranges. However, under low-speed conditions, their signal-to-noise ratio significantly degrades, resulting in extremely small or near-zero back EMF amplitudes. Additionally, they are susceptible to parameter mismatches and measurement noise, rendering back EMF-based position detection ineffective. To overcome the position estimation challenges of PMSMs under these conditions, researchers have proposed various high-frequency signal injection methods. Among these, the high-frequency injected salient pole tracking strategy does not rely on back-EMF information or precise motor parameters, enabling relatively stable estimation even at low speeds or during stationary operation [7]. Based on the form of injected signals and reference frame selection, high-frequency signal injection techniques are typically categorized into two main types: rotating high-frequency injection [8] and pulse-oscillation high-frequency injection [9]. Additionally, there exists a class of estimation methods based on fundamental wave models, including sliding mode observers, state observers, and Kalman filters. Although high-frequency signal injection methods demonstrate high accuracy and robustness in rotor position estimation, they involve substantial computational demands, require real-time capabilities, and feature complex implementation processes. They are prone to introducing additional electromagnetic noise and torque ripple, and their estimation performance remains limited under load variations and parameter perturbations [10,11]. Reference [12] indicates that methods based on zero-sequence voltage pulses exhibit insufficient estimation performance under low-wind-speed conditions. Reference [13] proposes a high-frequency pulsed square-wave voltage injection method based on an improved current signal extraction strategy, explicitly utilizing the injected high-frequency carrier voltage frequency as the system’s operating frequency for position detection and control functions. However, the high-frequency rotating injection method is suitable for motors with significant inherent spatial characteristics but not for surface-mounted permanent magnet synchronous motors. In [14], high-frequency pulsed voltage injection technology is combined with position observers or extended state observers to derive rotor position from q-axis current responses. Nevertheless, observer parameters significantly impact estimation results. Under special conditions, rotor position can also be detected without utilizing stator inductance variations. Reference [15] proposes demodulating the amplitude of three-phase currents using a recursive discrete Fourier transform. This method effectively avoids the delay issues caused by filter usage and digital control in traditional approaches but introduces additional hardware computational demands. Rotor position estimation methods based on voltage equations face an unavoidable limitation: when stator winding electrical parameters deviate significantly due to environmental changes or operational fluctuations, position recognition accuracy deteriorates markedly. Reference [16] detects the rotor’s initial position by solving the dynamic equations of the PMSM and estimates the rotor position based on vibration signals. This method exhibits low sensitivity to parameter variations. However, the approach detects vibrations at three specific positions, which affects estimation accuracy and increases computational effort.

With the advancement of multiphysics modeling and sensing technologies, utilizing vibration and structural response signals generated during motor operation for condition monitoring has gained increasing attention. Existing research indicates that the air gap electromagnetic force, serving as a critical link between electromagnetic and structural fields, excites specific frequency and modal characteristics of structural vibration in the stator. Its response properties are closely correlated with the motor’s operational state [17,18]. Analysis methods based on vibration or acoustic signals have been widely applied in motor fault diagnosis and condition monitoring, validating the feasibility of extracting rich operational information from non-electromagnetic signals [19,20].

Building upon this foundation, some scholars have begun to focus on the quantitative relationship between electromagnetic vibrations and operating conditions, analyzing the electromagnetic vibration characteristics of PMSMs under different operating conditions [21,22]. These studies provide theoretical foundations for utilizing structural vibration information to reflect internal electromagnetic states. However, existing work predominantly focuses on vibration characteristic analysis or fault identification, with relatively limited research on the formation mechanism of rotor position information within structural vibration modes and its application in sensorless control. Furthermore, the stability and applicability of these methods under conditions of significant noise interference and parameter variations require further validation [23,24].

To address these issues, this paper analyzes the sensitivity mechanism of structural vibration mode phase to rotor spatial position from the perspective of air gap electromagnetic force excitation coupled with stator structural vibration modes. Based on this, a rotor position estimation method based on structural vibration characteristics is proposed. By reasonably selecting vibration modes sensitive to rotor position and constructing corresponding signal processing and position calculation workflows, stable rotor position estimation under low-speed operating conditions is achieved. Experimental results demonstrate that the proposed method exhibits excellent position estimation accuracy under low-speed operating conditions, offering advantages such as simplicity, robustness, and high real-time performance. This paper focuses on revealing the physical mechanism and demonstrating a simulation-based proof-of-concept using coupled electromagnetic–structural FEA and signal-processing evaluation. Comprehensive hardware testbench validation with real accelerometer measurements and independent rotor angle sensing will be conducted in future work.

## 2. Materials and Methods

The vibration channels in PMSMs are primarily excited by electromagnetic force waves. These force waves originate from magnetic field harmonics in the air gap, which in turn induce periodic variations in Maxwell’s electromagnetic force. The electromagnetic force is transmitted through various structural components within the motor, ultimately manifesting as mechanical vibrations in the form of acceleration or displacement on the stator housing surface.

The detailed derivation is retained for completeness, while readers primarily interested in the estimation algorithm may proceed directly to Section 5.

### 2.1. Control System Architecture Based on Vibration Signal Detection

**Figure 1:**
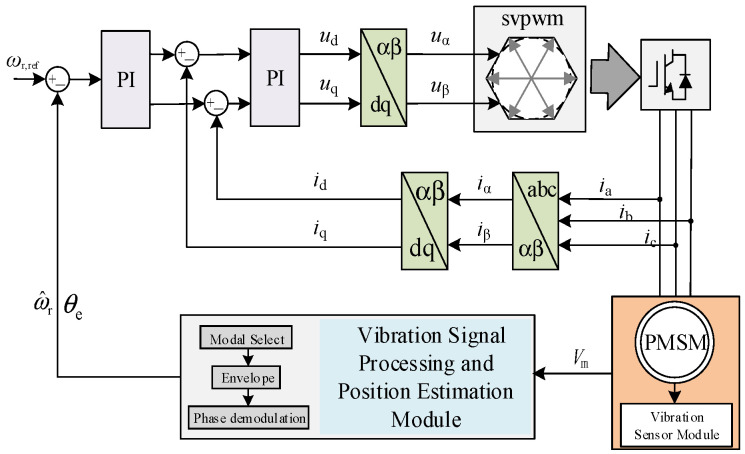
System structure block diagram.

The overall block diagram of the PMSM control system based on vibration signal detection is shown in Figure 1. This system comprises functional modules including the current loop, coordinate transformation, SVPWM modulation, inverter drive, vibration acquisition path, and data flow.

The proposed rotor position estimation method based on vibration signals is specifically designed for operating conditions where back-EMF information is extremely weak, such as during crawling low-speed operation, low bus frequency, strong electromagnetic interference, and significant load disturbances. Therefore, it can serve as an independent information channel parallel to traditional observation methods. Vibration sensors mounted on the stator frame housing transmit signals through conditioning circuits to the data acquisition unit. These signals then undergo a processing chain including modal gating, envelope demodulation, and phase back-calculation to ultimately yield the rotor electrical angle estimate. In actual zero-to-low-speed operation scenarios, this method serves as an observation tool for controllers under special conditions, collaborating with traditional control methods to enhance the system’s observation capabilities across all operating states. During actual operation, the controller can dynamically allocate weights or switch modes between traditional position observation methods and vibration-based methods based on operational status, self-diagnostic information, or specific operational requirements.

### 2.2. Nonlinear Saturation Characteristics of Stator Inductance

**Figure 2:**
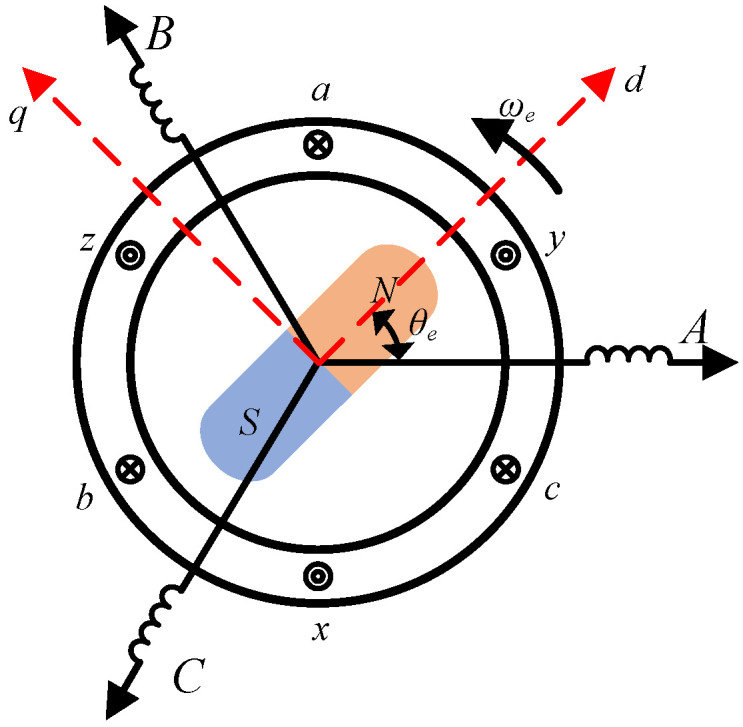
Simplified physical model of PMSM.

The simplified physical model of a PMSM is shown in Figure 2. The d-q axes form a rotating coordinate system, where the d-axis is coaxial with the rotor magnetic field and the q-axis is orthogonal to the d-axis. Points A, B, and C represent the axes of the three-phase stator windings a-x, b-y, and c-z, respectively. θe denotes the angle between the rotor d-axis and the A-axis, referred to as the rotor spatial position angle. ωe represents the rotor electrical angle of rotation.

The inductance of the PMSM stator winding is not a fixed constant but gradually decreases as magnetic flux and current increase, determined by the magnetic saturation characteristics of the core material. When a direct-axis current flows through the stator winding, the resulting magnetic potential either superimposes upon or cancels out the magnetic potential of the rotor permanent magnets, causing changes in the saturation level of the magnetic circuit. In surface-mounted permanent magnet synchronous motors, the rotor permanent magnets generate a constant d-axis magnetic flux. If the direction of the stator current aligns with the magnetic potential of the permanent magnets, the magnetic fluxes combine, increasing the magnetic flux density in the d-axis magnetic circuit and causing it to approach saturation. This results in a significant reduction in the equivalent d-axis inductance Ld compared to the unsaturated state. Conversely, if the stator current direction opposes the permanent magnet flux, the stator flux partially cancels the rotor flux. This reduces the total direct-axis flux and lowers the saturation level of the magnetic circuit, causing the direct-axis inductance to largely maintain its original high value. Therefore, Ld is essentially a nonlinear function of current, decreasing monotonically as the flux increases. Similarly, due to the spatial coupling of magnetic saturation, deep saturation in the direct axis also reduces the transverse axis inductance Lq through magnetic flux coupling via the transverse axis, a phenomenon termed transverse saturation of the magnetic circuit. This nonlinear effect not only impacts motor modeling accuracy but also provides a physical basis for subsequent rotor position detection and pole identification: by observing changes in stator inductance or current response under different magnetization levels, the direction of the permanent magnet’s north pole can be inferred.

**Figure 3:**
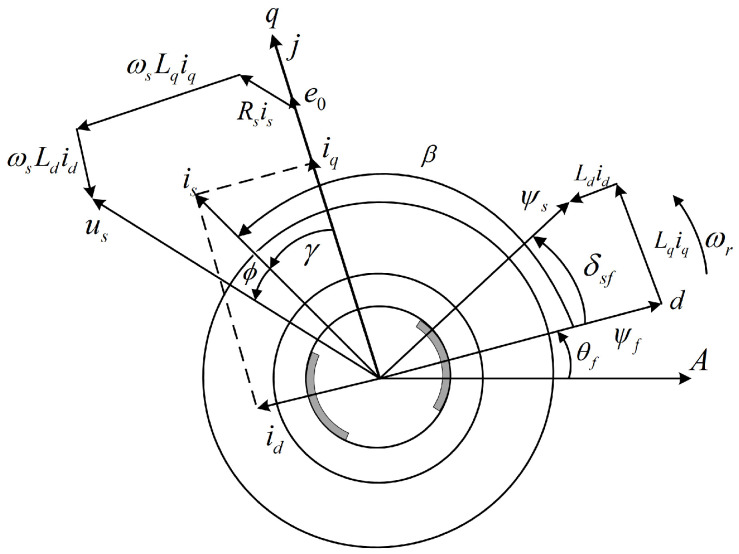
Schematic diagram of PMSM magnetomotive force vector.

The vector relationship of the magnetic flux linkage in the d–q coordinate system for a PMSM is shown in Figure 3. Here, the permanent magnet flux linkage, the stator direct-axis magnetic flux linkage, and the stator transverse-axis magnetic flux linkage form the composite magnetic flux linkage system. When the directions of the stator and rotor magnetic flux linkages are aligned, the composite magnetic flux linkage increases, and the magnetic circuit tends toward saturation. Conversely, when the directions are opposite, the composite magnetic flux linkage decreases, and the magnetic circuit tends toward linear behavior. This mechanism explains how magnetic flux linkage superposition and cancellation affect inductance variation.

**Figure 4:**
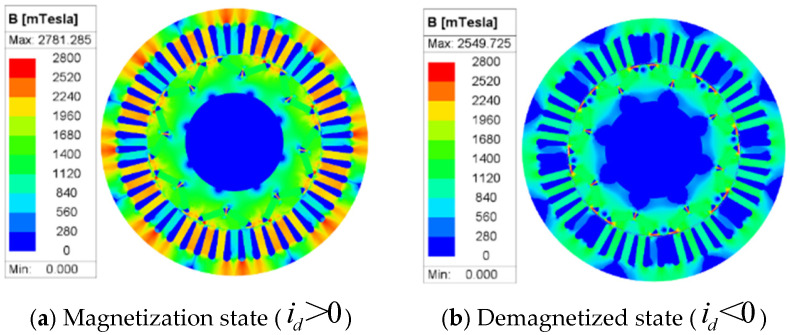
Magnetic flux density distribution of PMSM stator.

To further validate this analysis, a two-dimensional electromagnetic simulation of the motor’s magnetic field was conducted using ANSYS Maxwell 2024 R1. Figure 4 displays the stator magnetic flux density distribution under different currents. It can be observed that the magnetic flux density is significantly concentrated in the tooth-top region, with local flux exceeding 2.78 T, indicating that the magnetic circuit has entered a saturated state. In contrast, the flux distribution is more uniform, and the saturated region is significantly reduced.

**Figure 5:**
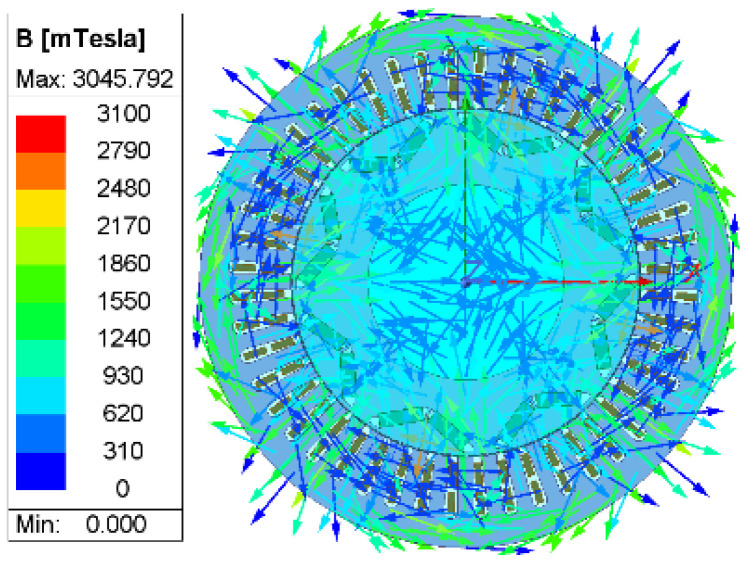
Magnetic flux direction distribution of PMSM.

The magnetic flux distribution of the PMSM is shown in Figure 5, illustrating the relationship between the stator and rotor magnetic flux directions. When the stator and rotor magnetic forces are in the same direction, the magnetic flux lines are dense and aligned. Conversely, when they are in opposite directions, the magnetic flux vectors exhibit partial reverse superposition zones, resulting in weakened magnetic flux density in the magnetic circuit. This finding further confirms the phenomena of local saturation and inductance reduction caused by magnetic force superposition.

The nonlinear saturation characteristics of stator inductance cause PMSMs to exhibit different inductance-flux linkage response relationships under varying magnetization states. Particularly for SPMSMs, within the linear region, the absence of significant salient pole effects makes it challenging to determine rotor position through inductance anisotropy. However, when considering saturation effects, the permanent magnet flux generates asymmetric magnetic permeability along the straight-axis direction, effectively forming virtual salient poles. This renders the motor sensitive to the direction of stator current, enabling initial position estimation and polarity identification based on inductance differences.

### 2.3. Mechanism of Electromagnetic Force Wave Formation

**Figure 6:**
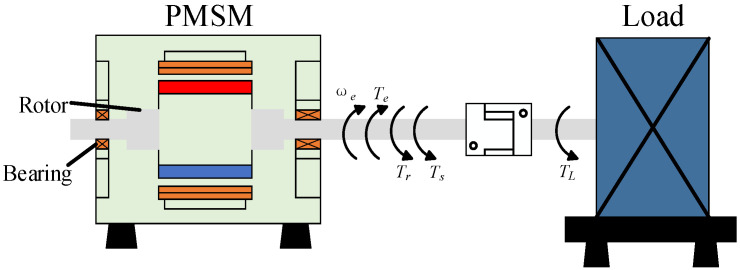
Schematic diagram of PMSM and load connection.

The schematic diagram of the PMSM connected to the load is shown in Figure 6. Its electromagnetic force primarily originates from the Maxwell stress tensor in the air gap, whose normal pressure can be approximated as:

(1) $$p(\theta ,t)=\frac{B_{g}^{2}(\theta ,t)}{2\mu_{0}}$$

In the equation: $B_{g}$ is the magnetic flux density in the air gap.

The air gap magnetic field is the superposition of the permanent magnet field and the stator armature field. Using Fourier expansion, it can be expressed as:

(2) $$B_{g}(\theta ,t)=\sum_{k}B_{m,k}\cos(kp\theta -\omega t-\phi_{m,k})+\sum_{n}B_{i,n}\cos(np\theta -\omega t+\theta_{e}(t)-\phi_{i,n})$$

In the equation, *p* denotes the pole number; k,n denotes the harmonic order; $\theta_{e}(t)$ denotes the electrical angle position; $B_{m,k},B_{i,n}$ represents the harmonic amplitude of the permanent magnet current.

Substituting the magnetic field harmonics into the pressure formula and expanding yields:

(3) $$p(\theta ,t)=\frac{1}{2\mu_{0}}{\left(B_{m}+B_{i}\right)}^{2}$$

It includes permanent magnetic force waves, electric current force waves, and electromagnetic coupling force waves.

Permanent magnet harmonics originate from the self-multiplication term of the permanent magnet field, with an order twice that of the permanent magnet harmonic order. They are independent of current and remain constant regardless of rotor angular velocity. Their expression is:

(4) $$p_{pm}(\theta )=\sum_{k}P_{pm,k}\cos(2kp\theta -2\phi_{m,k})$$

The current force wave originates from the self-term of the armature magnetic field, is influenced by the current frequency, and exhibits higher-order harmonics during high-frequency injection. Its expression is:

(5) $$p_{i}(\theta )=\sum_{n}P_{i,n}\cos(2np\theta -2\phi_{i,n})$$

The electromagnetic coupling force wave contains the rotor position angle, and rotor position information is primarily modulated into the vibration signal through this coupling term. Its expression is:

(6) $$p_{int}(\theta ,t)=\sum_{k,n}P_{k,n}\cos[(k-n)p\theta -\omega t+\theta_{e}(t)-(\phi_{m,k}-\phi_{i,n})]$$

### 2.4. Mechanism of Radial Vibration in Force-Wave-Driven Rotors

Electromagnetic force waves exhibit a sinusoidal distribution circumferentially along the air gap, acting upon the stator inner wall to generate circumferential and radial vibration mode excitation. The spatial order of the force waves is:

(7) m=|k−n|p

Force waves with *m = p* or 2*p* most readily excite the low-order modes of the motor housing, constituting the most prominent component in vibration measurements.

The dynamic equation for radial vibration response is:

(8) $$M\ddot{x}(t)+C\dot{x}(t)+Kx(t)=F_{m}(\theta_{e},t)$$

In the equation, *M*, *C*, and *K* represent the mass, damping, and stiffness matrices; x(t) is the node displacement vector; $F_{m}$ is the excitation corresponding to the force wave of the same order.

From Equation (8), it follows that as long as the force wave contains a modulation component corresponding to the rotor position angle $\theta_{e}$, the vibration output will necessarily include rotor position information.

## 3. Vibration Response Analysis Model Construction

### 3.1. Position Information Features in Vibration Signals

To facilitate understanding of the formation mechanism of rotor position information in vibration signals, the overall modeling process is presented prior to the analytical modeling derivation, as shown in Algorithm 1.
**Algorithm 1:** Analytical Modeling Process for Electromagnetic-Structure Coupled Vibration Response**Input:** $B_{g}(\theta ,t)$, p, $\omega_{e}$, $\mu_{0}$, Structural Modal Parameters $\left(\omega_{m},\zeta_{m},\Phi_{m}\right)$, sampled signal $x\left(t\right)$ or acceleration a(t) **Output:** Vibration phase $\varphi \left(t\right)$ Estimation of Rotor Electrical Angle $\theta_{e}(t)$ 1: Calculate Maxwell radial pressure: $p(\theta ,t)=\frac{B_{g}^{2}(\theta ,t)}{2\mu_{0}}$ 2: Perform a harmonic expansion of $B_{g}(\theta ,t)$ and substitute it into the pressure expression to obtain the pressure wave decomposition shown in Equation (9): $p(\theta ,t)=p_{pm}(\theta )+p_{i}(\theta ,t)+p_{int}(\theta ,t)$ 3: Initialization: Candidate order set M←∅ 4: for k=1:K do 5: for n=1:N do 6: Calculate the spatial order: m=∣k−n∣p 7: if m=p or m=2p then 8:  Add m to the candidate set: $M\leftarrow M\cup \left\{m\right\}$ 9: end if 10: end for 11: end for 12: Select the dominant structural modal order: $m^{⋆}=arg\;{max}_{m\in M}|H_{m}(\omega_{f})|$, where $H_{m}(\omega )$ is the modal transfer function of the mth order. 13: Construct equivalent radial electromagnetic excitation (corresponding to the primary-order force wave):            $F_{m^{⋆}}(t)=F_{0}cos(\omega_{f}t+m^{⋆}\theta_{e}(t)+\phi )$ 14: Establishing the modal dynamic equation of the $m^{⋆}$th order is shown in Equation (10): ${\ddot{q}}_{m^{⋆}}+2\zeta_{m^{⋆}}\omega_{m^{⋆}}{\dot{q}}_{m^{⋆}}+\omega_{m^{⋆}}^{2}q_{m^{⋆}}=K_{m^{⋆}}cos(\omega_{f}t+m^{⋆}\theta_{e}(t)+\phi )$ 15: Seeking steady-state response: $q_{m^{⋆}}(t)=A_{m^{⋆}}cos(\omega_{f}t+m^{⋆}\theta_{e}(t)+\phi_{0})$ 16: Mapped to measurement point vibration signals: $x(t)=\beta_{m^{⋆}}q_{m^{⋆}}(t)$ 17: Hilbert transform extracts the analytic signal: z(t)=x(t)+jH{x(t)} 18: Instantaneous phase: $\varphi (t)=arg(z(t))\approx \omega_{f}t+m^{⋆}\theta_{e}(t)+\phi_{0}$ 19: Estimation of Rotor Electrical Angle: $\theta_{e}(t)=\frac{\varphi (t)-\omega_{f}t-\phi_{0}}{m^{⋆}}$ 20: End

Based on the interaction term in the previous section, it follows from Equation (6) that the effective force wave containing position information can be expressed as:

(9) $$F_{pos}(t)=A\cos(\omega_{f}t+m\theta_{e}(t)+\phi )$$

Through the structural transfer function, the vibration output can be obtained:

(10) $$x(t)=|H(\omega_{f})|A\cos(\omega_{f}t+m\theta_{e}(t)+\phi +∠H)$$

It can be seen that the vibration signal phase contains $m\theta_{e}(t)$, and the vibration amplitude is modulated by the rotor angle. Even at extremely low speeds, or during zero-speed startup, the force wave still contains usable rotor position information.

### 3.2. Derivation of Analytical Expressions for Electromagnetic Force Density

The air gap magnetic field results from the superposition of the permanent magnet field and the armature field. According to the magnetomotive force method, the air gap magnetic flux density can be expressed as:

(11) $$B_{g}(\theta ,t)=B_{m}(\theta )+B_{i}(\theta ,t)$$

Its Fourier series expansion is:

(12) $$B_{m}(\theta )=\sum_{k=1}^{\infty }B_{m,k}\cos(kp\theta -\phi_{m,k})$$

(13) $$B_{i}(\theta ,t)=\sum_{n=1}^{\infty }B_{i,n}\cos(np\theta -\omega t+\theta_{e}(t)-\phi_{i,n})$$

In the equation, *k* and *n* denote the permanent magnet and current harmonic order indices, respectively; *p* represents the number of pole pairs; and denotes the electrical angle.

Maxwell pressure forms electromagnetic force density, and the Maxwell pressure in the air gap is:

(14) $$p(\theta ,t)=\frac{B_{g}^{2}(\theta ,t)}{2\mu_{0}}$$

Substituting and expanding yields three types of force waves:

(15) $$p(\theta ,t)=p_{pm}+p_{i}+p_{int}$$

Of particular importance are the interactive items:

(16) $$p_{int}(\theta ,t)=\sum_{k,n}P_{k,n}\cos[(k-n)p\theta -\omega t+\theta_{e}(t)-\Delta \phi_{k,n}]$$

Whenever k≠n occurs, the spatial order $\left(k-n\right)p\theta$ must appear, simultaneously incorporating the position modulation component $\theta_{e}(t)$. Therefore, the interaction force wave serves as the fundamental source of the vibration signal carrying rotor position information.

Derivation of force wave orders: The spatial force wave order is defined by Equation (7). In an 8-pole PMSM with *p* = 4, *m* = *p* and 2p, i.e., *m* = 4 and *m* = 8 are the most observable orders in vibration measurements. For the primary order *m* = *p* containing position information, the force wave can directly serve as the excitation term in the structural dynamics equation:

(17) $$F_{m}(t)=F_{0}\cos(\omega_{f}t+m\theta_{e}(t)+\phi )$$

### 3.3. Analytical Structural Dynamics Model Driven by Force Waves

Electromagnetic forces act on the inner wall of the stator yoke, inducing radial displacement. After neglecting structural nonlinearity, the stator structure can be modeled as a multi-degree-of-freedom system, as shown in Equation (8), where $F_{m}$ represents the radial electromagnetic force. Since the primary focus is on motor vibration signals—typically manifested as acceleration at a single point on the motor housing—the system can be reduced to modal coordinates. The structural modes satisfy:

(18) $$K\Phi =M\Phi \Omega^{2}$$

In the equation, Φ denotes the modal matrix, and Ω represents the diagonal matrix of modal natural frequencies.

Based on modal orthogonality, the system can be transformed into:

(19) $${\ddot{q}}_{i}(t)+2\zeta_{i}\omega_{i}{\dot{q}}_{i}(t)+\omega_{i}^{2}q_{i}(t)=\phi_{i}^{T}F(t)$$

In the equation, $q_{i}(t)$ represents the generalized coordinate of the *i*-th mode, $\zeta_{i}$ denotes the damping ratio, and $\phi_{i}$ is the modal shape vector.

Substituting the force waveform carrying rotor position information yields:

(20) $$\phi_{i}^{T}F(t)=\sum_{m}\alpha_{i,m}F_{m}\cos(\omega_{f}t+m\theta_{e}(t)+\phi )$$

In the equation, $\alpha_{i,m}$ is the modal participation coefficient.

Because the spatial distribution corresponding to the modal order *m* significantly excites the mode matching its order, the primary modal order *i* = *m* is typically approximated as:

(21) $${\ddot{q}}_{m}(t)+2\zeta_{m}\omega_{m}{\dot{q}}_{m}(t)+\omega_{m}^{2}g_{m}(t)=K_{m}\cos(\omega_{f}t+m\theta_{e}(t)+\phi )$$

Its steady-state solution is:

(22) $$q_{m}(t)=A_{m}\cos(\omega_{f}t+m\theta_{e}(t)+\phi +∠H_{m})$$

Among them: $A_{m}=K_{m}/\sqrt{{\left(\omega_{m}^{2}-\omega_{f}^{2}\right)}^{2}+{\left(2\zeta_{m}\omega_{m}\omega_{f}\right)}^{2}}$, $H_{m}(j\omega )=1/(\omega_{m}^{2}-\omega^{2}+j2\zeta_{m}\omega_{m}\omega )$.

If the vibration response at the measurement point is $x(t)=\beta_{m}q_{m}(t)$, where $\beta_{m}$ is the amplitude of the modal shape at the measurement point. Therefore, the final motor vibration signal can be expressed as:

(23) $$x(t)=A\cos(\omega_{f}t+m\theta_{e}(t)+\phi_{0})$$

In the formula, the $m\theta_{e}(t)$ phase component can be used for angle estimation, while the amplitude modulation exhibits weak coupling and serves as an auxiliary feature. The high-frequency force wave operates independently of motor rotation, enabling functionality even at low or zero speeds.

### 3.4. Vibration Signal Analysis Expression and Position Mapping Relationship

Motor vibration signals are typically represented by housing acceleration a(t), which exhibits a second-order differential relationship with displacement:

(24) $$a(t)=-A{\left(\omega_{f}+m{\dot{\theta }}_{e}(t)\right)}^{2}\cos(\omega_{f}t+m\theta_{e}(t)+\phi_{0})$$

If ${\dot{\theta }}_{e}(t)\approx 0$ during low-speed or startup phases, then:

(25) $$a(t)\approx -A\omega_{f}^{2}\cos(\omega_{f}t+m\theta_{e}(t)+\phi_{0})$$

Using the Hilbert transform, the instantaneous phase is obtained as:

(26) $$\varphi (t)=\omega_{f}t+m\theta_{e}(t)+\phi_{0}$$

Thus, the analytical mapping relationship between the vibration signal and the electrical angle is derived as:

(27) $$\theta_{e}(t)=\frac{\varphi (t)-\omega_{f}t-\phi_{0}}{m}=\frac{∠\left[x(t)+jH\{x(t)\}\right]-\omega_{f}t-\phi_{0}}{m}$$

## 4. Structural Vibration Simulation and Finite Element Verification

### 4.1. Electromagnetic Field Simulation Modeling and Electromagnetic Force Extraction

A 48-slot, 8-pole embedded PMSM was selected as the research subject, with specific parameters detailed in Table 1. Simulation employed the ANSYS Maxwell 2D transient electromagnetic field solver to model the air gap magnetic field and electromagnetic force density of the PMSM.

**Figure 7:**
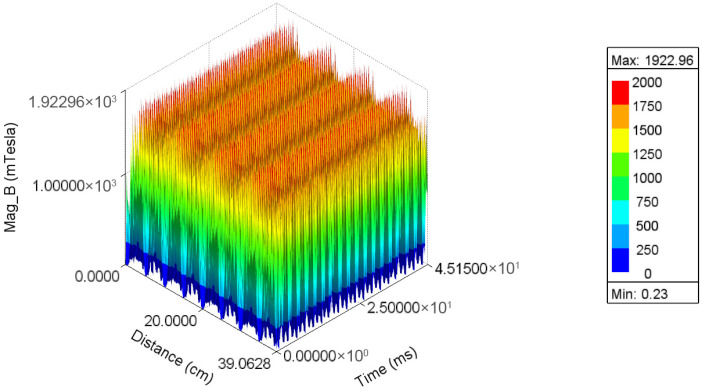
Spatiotemporal distribution map of the air-gap magnetic flux density.

To observe the spatiotemporal coupling characteristics of the air gap magnetic flux density in PMSMs holistically, its three-dimensional spatiotemporal distribution is shown in Figure 7. It can be seen that the magnetic flux density distribution is jointly influenced by the number of permanent magnet pole pairs and the number of stator slots, exhibiting distinct periodic spatial fluctuations corresponding to the harmonic model of the air gap magnetic field.

**Figure 8:**
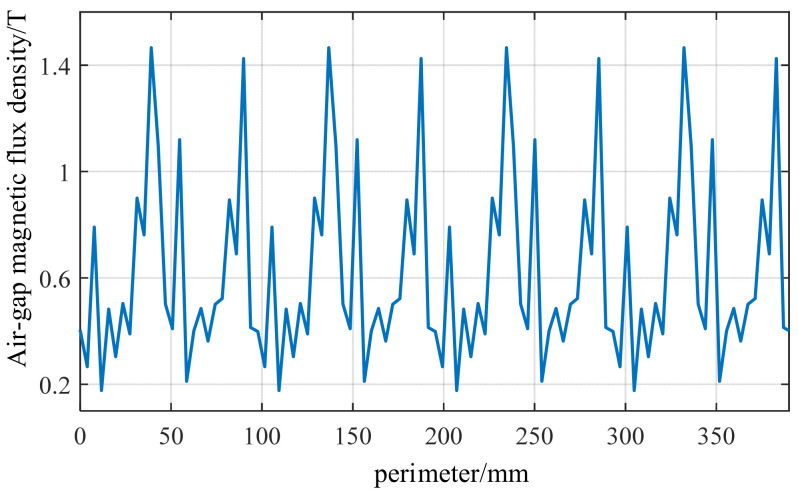
Spatial distribution map of the air-gap magnetic flux density.

To more precisely analyze the circumferential variation pattern of the air gap magnetic flux density, the magnetic flux density at a fixed moment was captured and plotted as a circumferential curve, as shown in Figure 8. The main magnetic flux density exhibits an 8-pole waveform resembling a sine wave. The superimposed high-frequency harmonic sawtooth-like local peaks generated by the 48 slots result in a non-sinusoidal air gap magnetic flux density waveform, with a maximum magnetic flux density of approximately 1.8–2.0 T.

**Figure 9:**
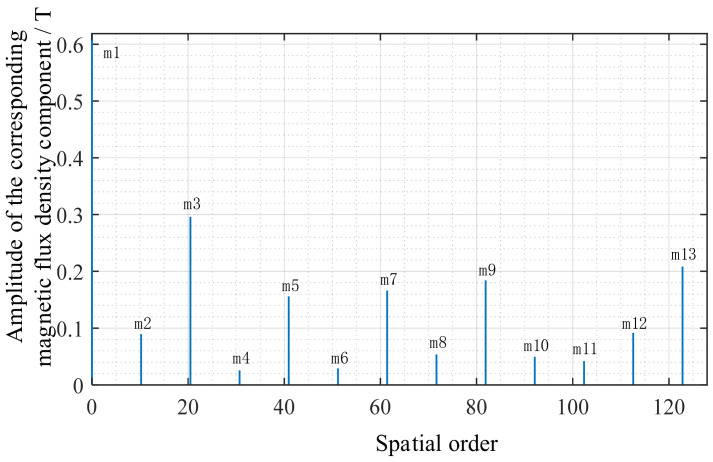
Fourier decomposition diagram of the air-gap magnetic flux density.

Figure 8 reflects both the permanent magnet harmonics and the slot effect, serving as direct input for constructing Maxwell force density and FFT analysis. To quantitatively analyze the harmonic components of magnetic flux density and verify whether the air gap magnetic flux contains harmonics such as *k* = 1, 3, 5, 7… as derived earlier, the magnetic flux waveform in Figure 8 undergoes FFT processing, yielding Figure 9. It can be observed that the harmonics with *m* = 1 and *m* = 3 exhibit the highest amplitudes, while the series of harmonics *m* = 5, 7, 9, 11, 13… gradually diminish in strength. Although the *m* = 1 and *m* = 3 harmonics are the strongest in the magnetic flux FFT, they do not carry rotor position information. The primary reason is that the vibration force wave originates from the magnetic flux square term B^2^(θ) and combines according to Equation (17) to form an interaction force wave. Therefore, the main peak of the air gap magnetic flux FFT corresponds to the main order of the electromagnetic force. In the vibration force wave, the harmonic that truly carries rotor position information is *m* = *p* = 4.

After obtaining the air gap magnetic flux density, radial and tangential force densities are calculated based on the Maxwell stress tensor. The force density expressions are:

(28) $$\left\{\begin{array}{l} F_{r}(\theta ,t)=\frac{B_{r}^{2}(\theta ,t)-B_{t}^{2}(\theta ,t)}{2\mu_{0}}\\ F_{t}(\theta ,t)=\frac{B_{r}(\theta ,t)B_{t}(\theta ,t)}{\mu_{0}} \end{array}\right.$$

**Figure 10:**
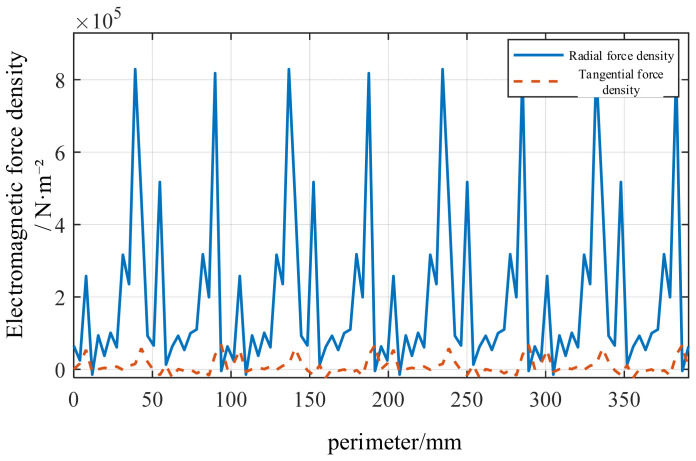
Distribution diagram of the radial and tangential air-gap electromagnetic forces.

Selecting the inner circumferential node of the stator and extracting the force density along the entire circumference yields Figure 10, which depicts the circumferential distribution of radial and tangential electromagnetic forces in the air gap. Since the radial Maxwell pressure serves as the primary excitation source for structural vibration, Figure 10 reveals that the radial force amplitude significantly exceeds that of the tangential force, with multiple slot harmonics superimposed within it.

**Figure 11:**
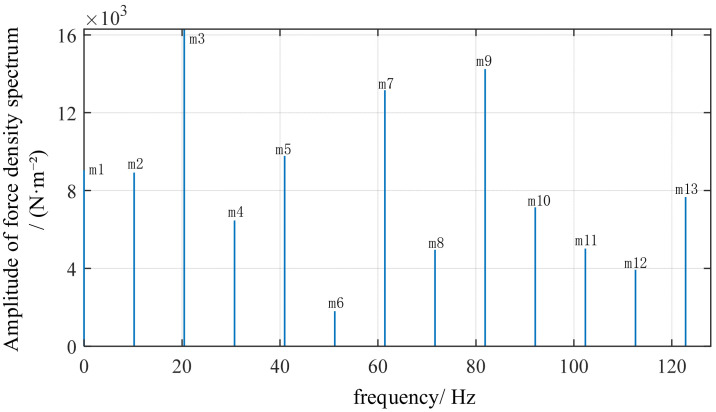
Fourier decomposition diagram of the radial electromagnetic force.

**Figure 12:**
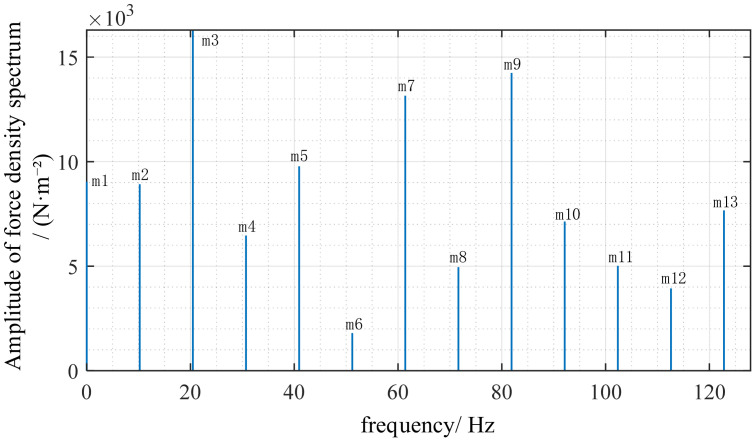
Fourier decomposition diagram of the tangential electromagnetic force.

To verify whether the order of force waves satisfies theoretical derivation, this section performs FFT analysis on the electromagnetic force density. FFT analysis of the electromagnetic force density curve in Figure 10 yields Figure 11 and Figure 12. The radial electromagnetic force density Fourier decomposition in Figure 11 shows that the *m* = 3 peak is the highest, followed by *m* = 1, caused by stator slot harmonics and magnetic circuit discontinuity. However, the *m* = 4 peak is distinctly present. This represents the primary force wave generated by the interaction between the permanent magnet field and the armature field, and it is the only force wave carrying rotor position information. In the tangential force density Fourier decomposition shown in Figure 12, the overall amplitude of the tangential force is smaller than that of the radial force. The *m* = 4 peak still exists but with a lower amplitude. Therefore, in the harmonic response, the tangential force has a weaker influence and acts only as a secondary excitation. Spectral lines are primarily concentrated in low-order slot-related harmonics. Although slot harmonics are typically stronger, they do not induce position modulation and do not significantly affect the primary vibration mode, thus not compromising the method’s validity.

### 4.2. Stator Structure Modal Analysis

**Figure 13:**
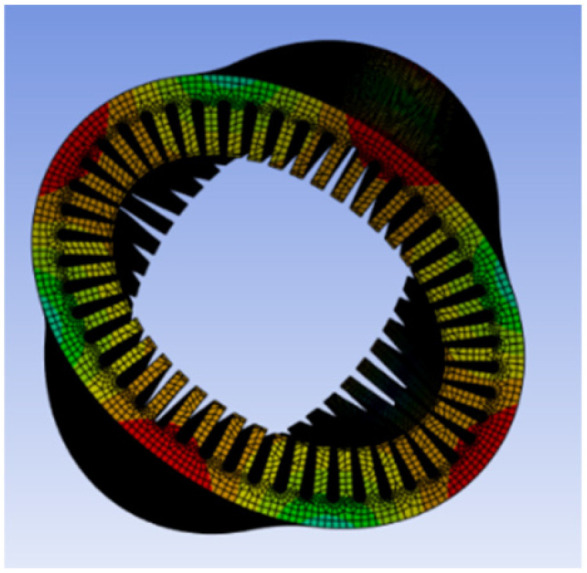
Second-order mode at 1183.9 Hz.

**Figure 14:**
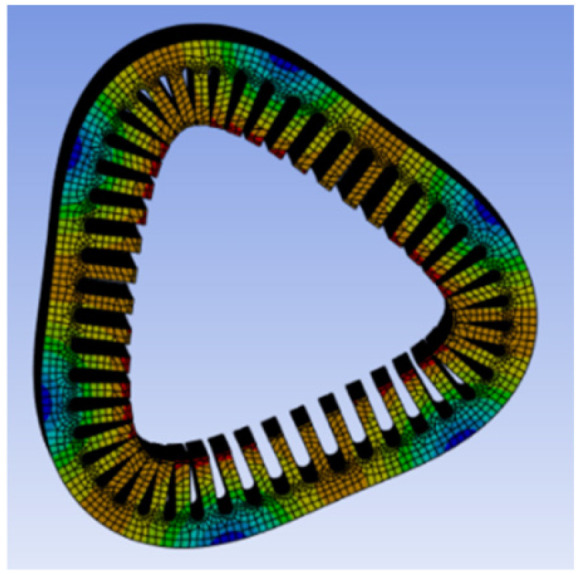
Third-order mode at 1562.5 Hz.

**Figure 15:**
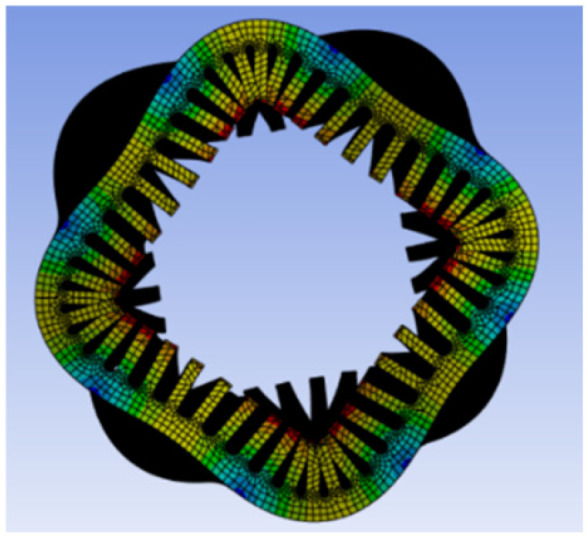
Fourth-order mode at 3907.8 Hz.

**Figure 16:**
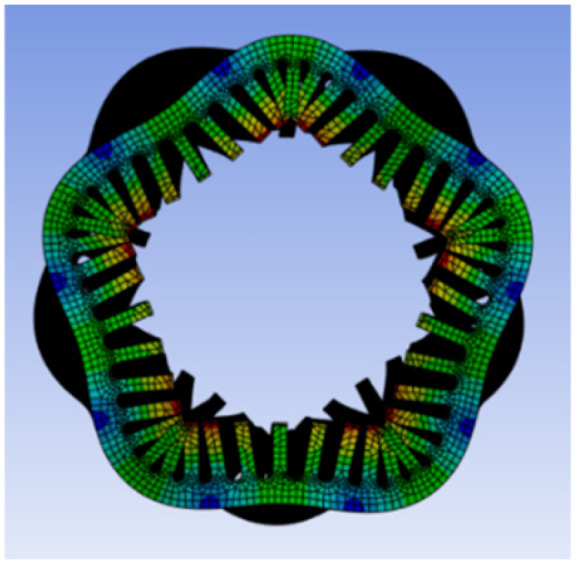
Fifth-order mode at 5203.9 Hz.

To investigate the coupling mechanism between electromagnetic force waves and the structural vibration response of the motor, and to verify whether the vibration response is dominated by the mth-order force wave, a modal analysis of the PMSM stator housing structure is required. This analysis aims to determine its natural vibration modes, spatial order, and natural frequency distribution. This analysis employs ANSYS Mechanical to perform modal solutions on the actual PMSM structure, systematically verifying the simulation results against the aforementioned theoretical derivation. The low-order modal shapes are shown in Figure 13, Figure 14, Figure 15 and Figure 16.

As shown in the figure above, the PMSM stator housing system exhibits a series of natural modal forms with typical spatial lobular characteristics. Among these, the second-order mode in Figure 13 presents an elliptical deformation structure caused by geometric machining errors or assembly asymmetry, but it does not align with the primary harmonic components of the electromagnetic force. Figure 14 shows the third-order mode with a three-lobed vibration pattern, corresponding to the *m* = 3 strong harmonic in the air gap magnetic flux density and force density FFT. However, since this order does not carry electrical angle information, its significant vibration participation does not affect position estimation. Figure 15 shows a fourth-order mode exhibiting a standard four-lobed deformation. This is the key mode consistent with the *m* = 4 electromagnetic interaction force wave order, possessing a natural frequency of 3907.8 Hz. Its spatial order matches the *m* = 4 force wave, making it the primary carrier of $4\theta_{e}$ phase modulation in the vibration signal. Figure 16 depicts a fifth-order mode representing a higher-order vibration.

## 5. Design of Vibration Signal Processing Methods

### 5.1. Modal Selection Criteria

The effectiveness of the proposed vibration-phase-based position estimation relies on the selection of a structural mode that can stably carry the rotor position modulation. In this work, the modal selection is not based on a global optimal search but follows a physics-guided and practically observable criterion. The dominant mode is determined according to the following three principles.

First, spatial order consistency between the electromagnetic interaction force wave and the structural mode is required. As derived in Section 3, the interaction force component with spatial order *m* = *p* is the only component that contains rotor position information. Therefore, the structural mode with the same spatial order is the primary candidate for modal gating.

Second, the selected mode should exhibit dominant modal participation within the measured frequency band. This ensures that the vibration response is mainly contributed by the target mode and provides sufficient signal-to-noise ratio for phase demodulation.

Third, the mode must be observable at feasible sensor locations. In practice, the vibration sensor cannot be placed arbitrarily at the theoretical maximum response point. Therefore, the selected mode should present a non-zero and sufficiently large modal amplitude at accessible positions on the stator housing.

Based on the above criteria, the fourth-order structural mode is selected for the studied 48-slot/8-pole machine. This mode matches the spatial order of the interaction force wave (*m* = 4), shows dominant energy in the corresponding band, and exhibits clear observability on the stator frame.

#### 5.1.1. Robustness Boundary and Practical Considerations

In practical machines, vibration measurements can be influenced by multiple sources, such as bearing-related vibration, fan and cooling excitation, torque ripple, and mounting boundary variations. The proposed method mitigates these influences primarily through modal gating by extracting a band-pass component around the target mode and through phase-based feature extraction, which is less sensitive to broadband amplitude contamination.

Nevertheless, if a strong interference component appears within or close to the selected modal band, or if structural parameters drift significantly due to severe bearing degradation or mounting looseness, the observability and signal-to-noise ratio of the target mode may be reduced. In such cases, adaptive band tracking, multi-sensor fusion, and online mode re-identification can be employed to enhance robustness. These aspects define the current robustness boundary and will be further investigated in future work.

It should be noted that the present validation uses synthetic noise superimposed on FEA-derived vibration signals, which cannot fully reproduce all mechanical coupling effects in real machines. A hardware testbench with real vibration measurements under practical operating conditions is currently under development and will be reported in future work.

#### 5.1.2. Temperature-Induced Modal Drift

Temperature variation may change the stiffness and damping of the stator structure, leading to a shift in the natural frequency of the selected mode. However, such thermal effects mainly influence the resonance frequency rather than the spatial order of the mode or the underlying force–mode coupling mechanism.

The proposed method extracts rotor position information from the phase modulation of the band-pass signal after removing the linear carrier trend. Therefore, it does not rely on an exact fixed resonance frequency. In practice, moderate temperature-induced frequency drift can be accommodated by using a sufficiently wide band-pass window or by adopting an adaptive center-frequency tracking strategy.

It should be noted that extreme thermal conditions may reduce the modal observability and affect the signal-to-noise ratio. The development of an adaptive modal tracking method under wide temperature variation will be investigated in future work.

### 5.2. Vibration Signal Preprocessing and Modal Gating

The operating environment of electric motors is complex, so motor vibration signals typically contain multiple superimposed components, including electromagnetic radial force excitation, inherent modal responses of the stator and frame, and electromagnetic and mechanical impact noise. Vibration sensors are typically mounted on the stator frame housing. Vibration acceleration signals are captured via accelerometers, amplified and conditioned, then fed into the AD conversion channel. To ensure sampling stability and feature capture capability, the sampling frequency must satisfy the bandpass processing requirements for the previously verified fourth-order natural modal frequency. Therefore, $f_{s}\geq 10f_{m}\approx 40\mathrm{kHz}$ is adopted.

Raw vibration signals typically contain DC bias and trend components that compromise the stability of envelope extraction. Therefore, preprocessing steps including mean removal, trend removal, and signal amplitude normalization—are essential to ensure consistency across different batches of data. These treatments preserve the spectral structure of the vibration signals while significantly enhancing the robustness of subsequent filtering and demodulation processes.

**Figure 17:**
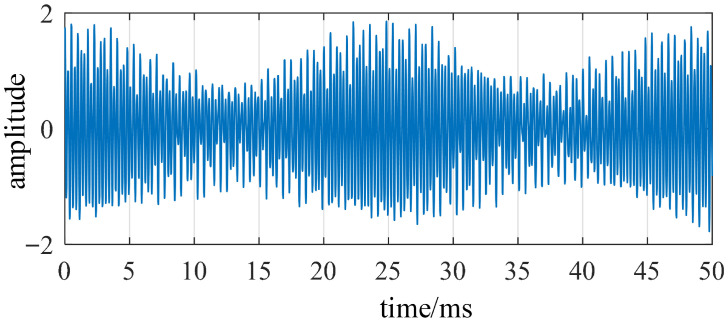
Original vibration signal.

**Figure 18:**
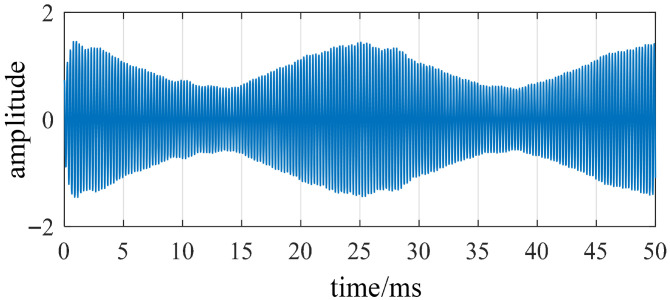
Filtered vibration signal.

Finite-element simulation results indicate that the Fourier singularity of the radial electromagnetic force is most pronounced at the fourth order, corresponding to the fourth-order mode of the stator structure. This mode is the only component in the vibration signal exhibiting amplitude modulation characteristics that vary with electrical phase angle. Therefore, a bandpass filter must be employed to extract the vibration response in this frequency band, ensuring that the vibration energy of the target mode is fully captured even under disturbance conditions. Figure 17 illustrates the typical signal changes before and after modal gating. The filtered vibration signal obtained after band-pass processing is shown in Figure 18. After bandpass processing, the signal exhibits regular high-frequency carrier vibrations with an envelope slowly modulated by the electrical angle.

### 5.3. Vibration Signal Feature Extraction Methods

The processed vibration signal can be approximated as:

(29) $$x(t)=A(t)\cos(2\pi f_{m}t+\phi (t))$$

In the equation: the envelope is A(t)=|z(t)|; the phase is ϕ(t)=arg(z(t)).

**Figure 19:**
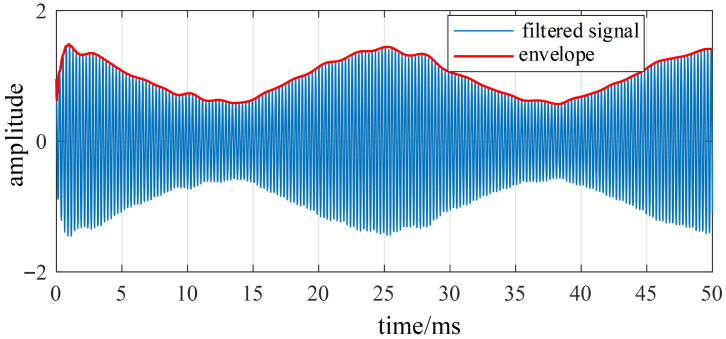
Hilbert envelope of the vibration signal.

The envelope diagram shown in Figure 19 reveals the gradual undulating energy changes over time, corresponding to the quadruple-frequency characteristics of the rotor electrical angle derived in the preceding theoretical analysis.

**Figure 20:**
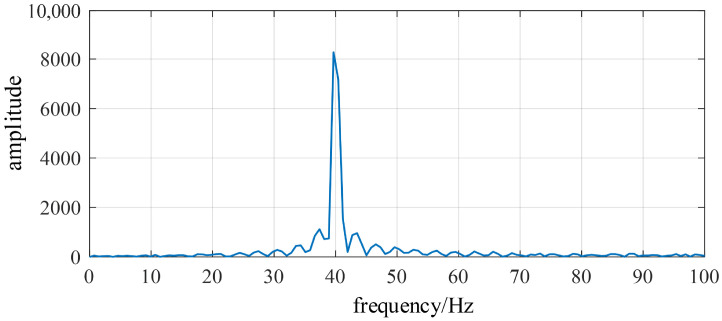
Envelope spectrum of the vibration signal.

The rotor angular position can be estimated through the phase variation of the envelope peak. Performing an FFT on the envelope yields the envelope spectrum shown in Figure 20. It can be observed that the spectral peak occurs at 40 Hz, corresponding to the mechanical rotational frequency and the $4f_{e}$ harmonic, consistent with the analytical model prediction. This further validates the feasibility of rotor position estimation based on vibration signals.

This vibration signal processing workflow uses the stator structural vibration response extracted from finite element simulation as input. Its instantaneous phase is calculated using Equation (27) and mapped to the rotor electrical angle via Equation (28). By comparing the rotor angle derived from the vibration phase calculation with the rotor angle trend under the corresponding operating condition in the finite element simulation, the results demonstrate excellent consistency between the two, with a maximum angular deviation of less than 0.02 rad.

After completing modal selection and signal analysis construction, the instantaneous phase of the carrier signal can be regarded as the superposition of two components: the high-frequency natural vibration of the stator near the fourth-order natural mode, and the slow modulation caused by the change in electrical angle due to electromagnetic forces. The high-frequency inherent vibration of the stator near the fourth-order natural mode manifests as a primary trend of phase rising approximately linearly with time, reflecting the inherent dynamic characteristics of the motor structure itself. The latter component represents the manifestation of electrical angle information within the vibration channel, directly corresponding to the aforementioned coupling mechanism between force wave excitation and the fourth-order mode response.

**Figure 21:**
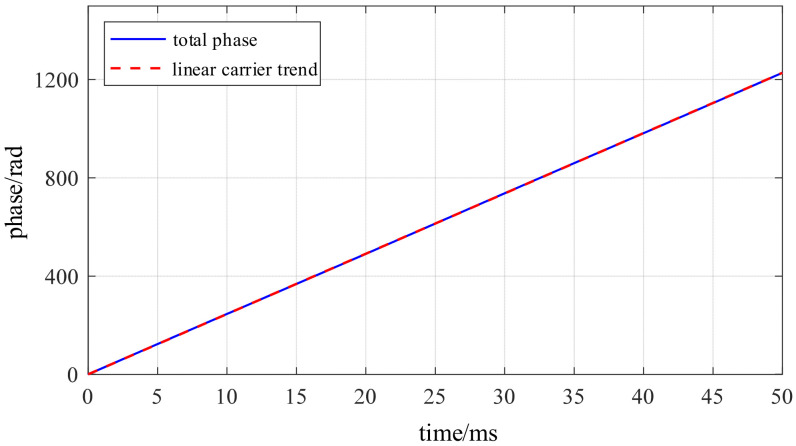
Schematic diagram of the analytic phase of a bandpass signal.

**Figure 22:**
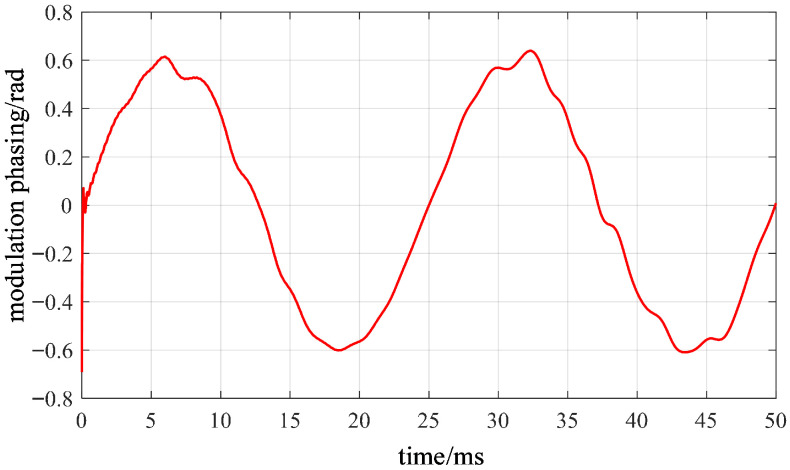
Demodulated and phase-locked waveform after carrier removal.

The schematic diagram of the phase analysis of the carrier signal is shown in Figure 21. It can be observed that the phase exhibits overall linear growth, with its slope corresponding to the modal natural frequency. The superimposed slow fluctuations, however, vary periodically with the electrical angle. Therefore, extracting electrical angle information from the vibration phase essentially involves discarding the high-frequency natural vibrations of the stator near the fourth-order natural mode while retaining the portion that varies with the electrical angle. Figure 22 presents the adjusted phase-modulated waveform after removing the linear trend. The periodic component corresponding to electrical angle variations is clearly visible. Following linear trend removal, the adjusted phase-modulated $\phi_{d}\left(t\right)$ exhibits periodic low-frequency oscillations. Its frequency is proportional to the electrical angular frequency, while its amplitude is determined by the structural electromagnetic coupling coefficient. This fully validates the mechanism described in the analytical model, where the modal phase slowly oscillates with electrical angle variations.

First, remove the linear phase components corresponding to the natural modes from the analyzed phase, allowing the remaining phase to primarily reflect changes induced by electromagnetic force modulation. Since the electromagnetic force in a 4-pole permanent magnet synchronous motor primarily manifests as a spatial order of *m* = 4, its modulated phase exhibits a clear linear mapping relationship with the electrical angle. Consequently, this modulated phase can be proportionally converted into an estimated electrical angle, enabling a direct mapping between vibration signals and rotor position. This method relies on explicit physical coupling relationships and is independent of back-EMF magnitude, thus offering significant advantages in zero-speed/low-speed regions and under strong electromagnetic interference conditions.

## 6. Rotor Position Estimation: FEA-Based Verification and Control-Level Evaluation

The experimental subject is an embedded permanent magnet synchronous motor, whose main parameters are shown in Table 1. The motor’s electromagnetic characteristics and stator structural vibration response were obtained through finite element simulation and served as inputs for vibration signal processing and rotor position calculation. The vibration response obtained from the electromagnetic–structural coupled FEA was used as the input of the signal-processing chain, with a sampling frequency set to 40 kHz to meet the sampling requirements for the primary structural modal frequencies. The control system was developed in the MATLAB/Simulink R2023b environment, incorporating a current loop, a speed loop, and a rotor position estimation module based on the vibration signals.

To strengthen the quantitative linkage between the analytical mechanism and verification results, this section summarizes how each theoretical claim is supported by the corresponding simulation-based evidence. The interaction electromagnetic force wave derived in Section 3 contains rotor position modulation and excites the structural mode with the same spatial order. After modal gating around the selected mode, the analytic phase of the band-pass vibration signal is computed and its carrier trend is removed to obtain the phase modulation component, which is mapped to the rotor electrical angle.

### 6.1. Steady-State Low-Speed Operating Condition Simulation Verification

**Figure 23:**
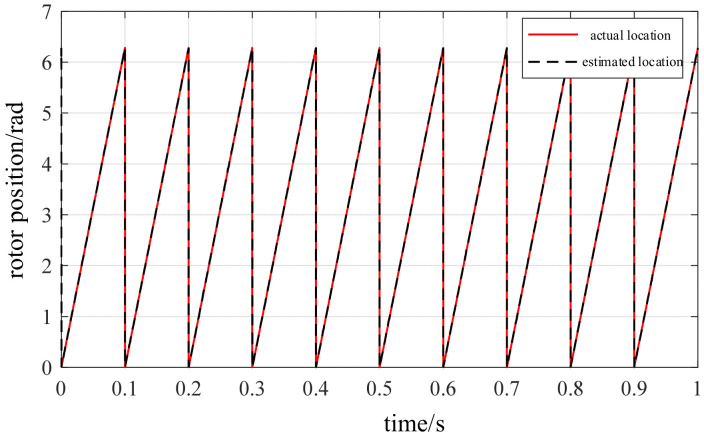
Estimated and actual rotor position.

**Figure 24:**
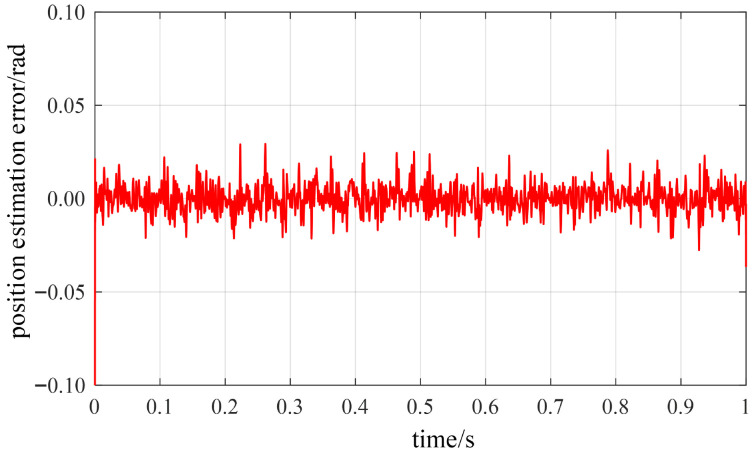
Rotor position estimation error.

Under steady-state low-speed operating conditions, the proposed rotor position estimation method based on structural vibration signals was validated. At a steady-state low speed of approximately 75 r/min, the estimated rotor position obtained through vibration channel demodulation is compared with the actual position in Figure 23, while the position estimation error curve is shown in Figure 24. The results demonstrate that the observed rotor position closely tracks the actual position trajectory with high precision, maintaining an overall error within ±0.02 rad. No significant systematic bias is observed during the steady-state phase, with deviations consistently suppressed within a minimal range. Quantitative analysis indicates that the root mean square error (RMSE) of the position estimation error is approximately 0.017 rad, with a maximum error of about 0.078 rad. Furthermore, the dynamic response process exhibits no significant overshoot or lag phenomena.

Throughout the entire test interval, the error curve exhibited no significant cumulative deviation or drift issues, demonstrating strong interference resistance and excellent stability. The simulation results indicate that the proposed rotor position estimation method based on vibration signal detection maintains stable phase resolution capabilities even under complex operating conditions such as high noise and strong electromagnetic interference, fully validating its reliability.

**Figure 25:**
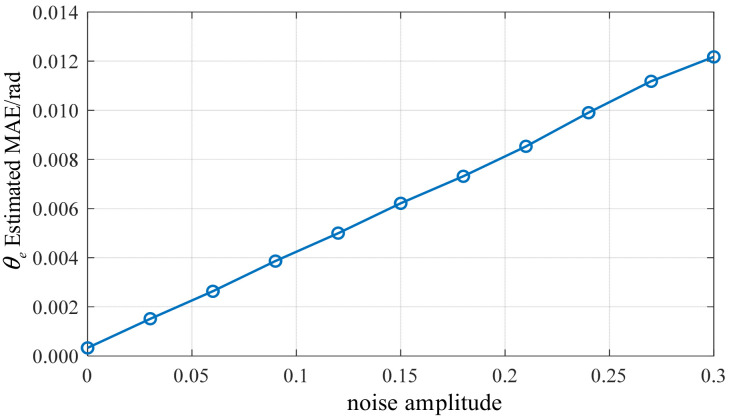
MAE curve of electric angle estimation under different noise amplitudes.

To further evaluate the robustness of the proposed method under complex background noise conditions, a systematic scan was conducted on vibration signals superimposed with noise of varying amplitudes. Ten trials were performed at each noise level, and the mean absolute error (MAE) was calculated and averaged, as shown in Figure 25. The MAE curve indicates that as noise amplitude increases, the algorithmic error rises approximately linearly. However, the overall level remains consistently controlled within 0.015 rad, demonstrating significantly superior performance compared to traditional low-speed position estimation methods based on electrical parameter feedback. The proposed method exhibits strong resistance to noise disturbances and demonstrating strong robustness against noise disturbances in the simulation environment. At zero and low speeds, many traditional observation methods based on back EMF suffer performance degradation due to excessively low voltage signal amplitudes. In contrast, the robustness of the vibration-based method demonstrates a distinct advantage in this operating range.

To assess the influence of sensor placement, a simulation-based observability evaluation was conducted based on the selected mode shape. Multiple feasible locations on the stator housing were considered. As the normalized modal amplitude decreases, the estimation error increases due to reduced effective SNR. However, stable estimation remains achievable as long as the selected mode is sufficiently observable. This indicates that the proposed approach does not depend on a unique optimal point, but allows flexible installation within modal high-response regions, as summarized in Table 2.

### 6.2. Simulation Verification of Variable-Speed Operating Conditions

**Figure 26:**
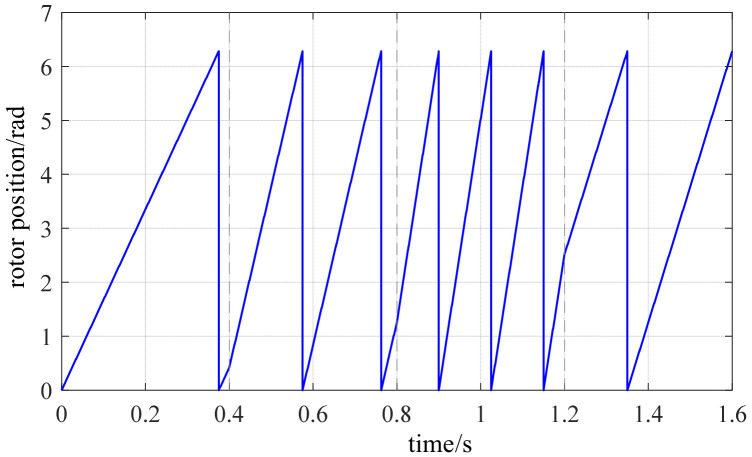
Actual rotor position waveform under speed change.

**Figure 27:**
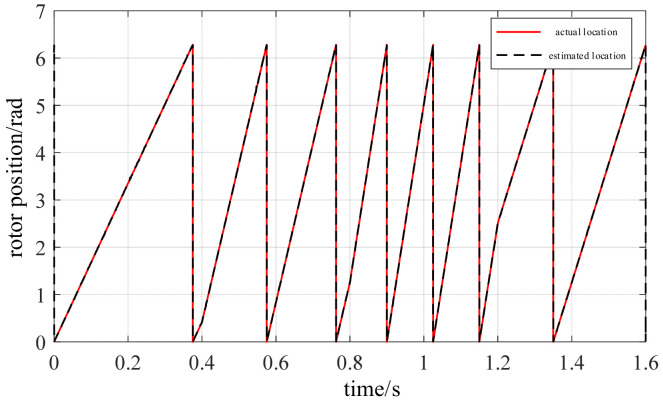
Estimated and actual rotor position under speed variation.

**Figure 28:**
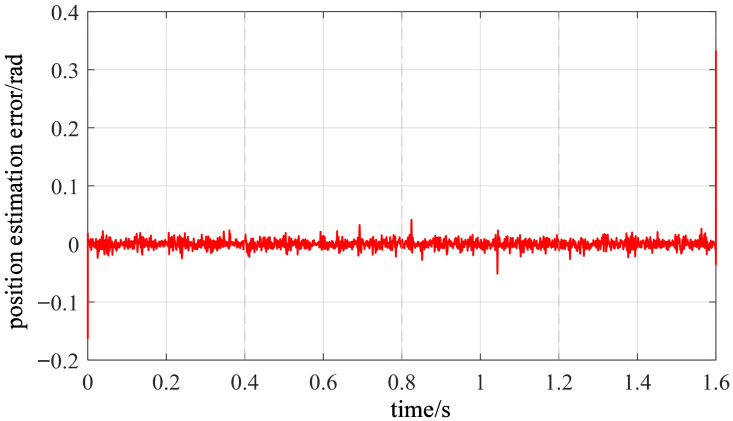
Rotor position estimation error under speed variation.

To further evaluate the adaptability of the vibration channel position estimation method during speed variations, a segmented variable-speed operating condition was established as shown in Figure 26. The PMSM operation comprises three phases: 75 r/min, 400 r/min, and deceleration to 150 r/min. The motor accelerated to 100 r/min within 0.4 s to complete the startup process and enter low-speed operation. At 0.4 s, it switched to 400 r/min and maintained a medium-speed steady state. Between 1.2 and 1.6 s, it decelerated again to 150 r/min. Figure 27 and Figure 28 show the electrical angle estimation tracking waveform and error curve for this operating condition, respectively.

Figure 27 shows the tracking results between the estimated rotor position and the actual value under dynamic variable-speed conditions. It can be observed that during acceleration, constant speed, and deceleration phases, the estimated curve consistently closely follows the actual rotor position changes without noticeable phase lag or distortion. Figure 28 displays the corresponding rotor position estimation error. Except for brief spikes at isolated moments caused by noise and mechanical shocks, the error remains within a small range for most of the time. Statistical results indicate that both the RMSE and MAE of this method are well below 0.05 rad under these conditions. This demonstrates that even during step changes in rotational speed, the vibration-based position estimation algorithm maintains good dynamic tracking capability and robustness.

### 6.3. Simulation Verification Under Load Disturbance Conditions

To further validate the robustness of the proposed vibration channel position estimation method under actual operating conditions and evaluate the performance of the entire sensorless starting process, this section designed a low-speed load disturbance experiment. During the experiment, the rotor position was initially set to $\theta_{r}=180{}^{\circ}$. The PMSM speed was gradually increased from 0 r/min to 180 r/min while applying a 2 N·m load disturbance to the motor. The actual rotor position, estimated position, q-axis current, and estimated error waveforms were recorded over time.

**Figure 29:**
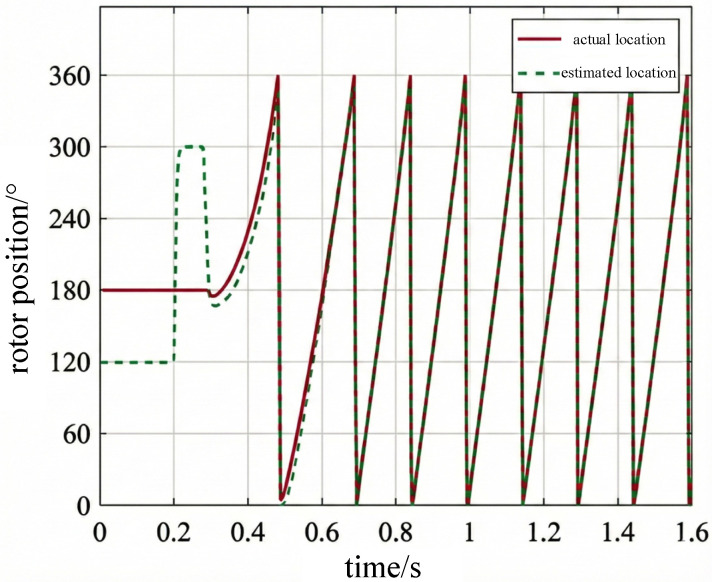
Comparison of actual and estimated rotor position under load disturbance.

As shown in Figure 29, when load is applied, the sudden increase in electromagnetic torque causes an instantaneous drop in electrical angular velocity, resulting in a slight phase lag in the actual position curve relative to the estimated position. The proposed vibration feature estimation method rapidly tracks this change, with the estimated curve exhibiting only a brief deviation within the disturbance interval before quickly recovering and realigning with the actual position, demonstrating excellent disturbance suppression capability.

**Figure 30:**
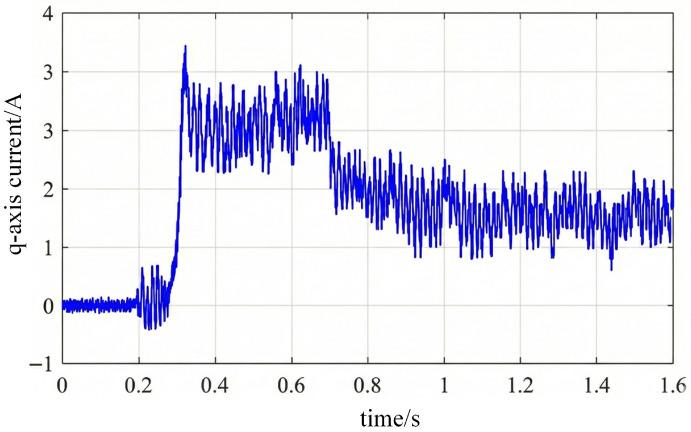
q-axis current response under load disturbance.

To verify the load variation process from the electromagnetic response perspective, Figure 30 shows the corresponding q-axis current waveform. At 0.2 s, the current rises rapidly, with its amplitude matching the system torque demand. The vibration channel introduced by the algorithm imposes no additional burden on the current loop, ensuring smooth and controllable current changes throughout the process.

**Figure 31:**
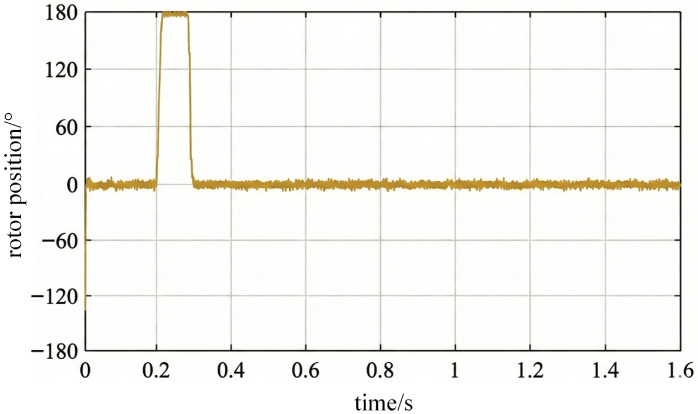
Rotor position estimation error under load disturbance.

Under the experimental conditions, when a sudden load torque change occurs, the rotor position estimation error exhibits a transient offset. This offset is primarily caused by short-term phase disturbances induced by the torque change. Figure 31 shows the position estimation error curve. It can be observed that the transient offset occurring during load application corresponds to the short-term phase disturbance caused by the torque jump. Subsequently, the error converges to the steady-state error range of ±3° within approximately 50 ms. This result demonstrates that the proposed method exhibits both rapid transient response capability and stable estimation accuracy when subjected to external load disturbances, reflecting excellent dynamic convergence characteristics and low-speed disturbance rejection performance.

A sudden load torque variation introduces a transient disturbance in the vibration phase due to short-term electromechanical dynamics. However, the proposed estimator is phase-modulation-based and does not rely on steady-state torque balance, hence explicit load compensation is not mandatory. As shown in Figure 31, the error exhibits a short-duration offset and rapidly converges back to the steady-state range. In future work, adaptive filtering and multi-sensor fusion will be considered to further suppress transient offsets under abrupt load steps.

The proposed method does not require additional voltage injection or iterative observers. Its main computations consist of band-pass filtering, analytic phase extraction, phase unwrapping, and linear trend removal, which are lightweight and suitable for real-time implementation. A structured comparison with representative back-EMF and HF injection methods is provided in Table 3. The computational burden of the proposed algorithm is low. Based on operation-count analysis, the computational burden of the proposed algorithm occupies only a small fraction of a typical control cycle, indicating good real-time feasibility for DSP/MCU implementation.

## 7. Conclusions

To address the issues of weak back-EMF signals and reduced estimation accuracy in traditional sensorless methods during low-speed operation of permanent magnet synchronous motors, this paper proposes a rotor position estimation method based on structural vibration characteristics. By analyzing the coupling relationship between air gap electromagnetic forces and stator structural vibration modes, the rotor position information embedded in specific vibration mode phases is revealed, and a corresponding vibration signal processing and position calculation workflow is established. Simulation results demonstrate that the proposed method achieves stable and continuous rotor position estimation within the low-speed operating range, even under noisy and load disturbance conditions. Its estimation accuracy significantly outperforms conventional sensorless methods based on back EMF, while exhibiting robust performance against variations in motor parameters and external disturbances. This method does not rely on back-EMF amplitude nor require additional high-frequency excitation signals. It can serve as an independent position information acquisition channel under low-speed conditions, offering a novel approach for sensorless control of permanent magnet synchronous motors at low speeds. It should be noted that the proposed method exhibits certain dependencies on the observability of structural vibration modes and sensor placement locations. Its generalizability across motors with different structural configurations warrants further investigation. Future work will integrate multi-information fusion strategies and experimental platform validation to extend the applicability of this method across a broader speed range.

## Figures and Tables

**Table 1:** Simulation parameters of the PMSM motor.

Fixed Parameters (Units)	Numerical Value
Stator inner/outer diameter (mm)	125/190
Rotor inner/outer diameter (mm)	70/123.8
Air gap length (mm)	0.6
Effective length (mm)	100
Number of stator slots	48
Number of pole pairs	4
Permanent magnet materials	NdFeB38UH
Rotational speed (rpm)	3000
Number of conductors per slot	8
Number of winding layers	Double-decker
Number of parallel branches	2

**Table 2:** Influence of sensor location on estimation accuracy (simulation-based observability evaluation).

Sensor Position (Mechanical Angle)	Normalized Modal Amplitude	RMSE (rad)	MAE (rad)
0°	1.00	0.017	0.015
45°	0.82	0.019	0.016
90°	0.65	0.024	0.020
135°	0.38	0.031	0.026

**Table 3:** Computational complexity and real-time feasibility comparison.

Method	Required Signals	Main Processing Blocks	Extra Excitation	Iterative Observer	Complexity Level	Real-Time Note
Back-EMF-based	Voltage & current	filtering + PLL/observer	No	Yes	Low	Good at medium/high speed
HF injection	Current	demodulation + filters + observer	Yes	Often yes	High	Higher burden, EMI/torque ripple
Proposed	Vibration acceleration	band-pass + analytic phase + trend removal	No	No	Medium	Efficient DSP/MCU implementation

## Data Availability

The original contributions presented in this study are included in the article. Further inquiries can be directed to the corresponding author.
